# Lignan Glycosides and Flavonoid Glycosides from the Aerial Portion of *Lespedeza cuneata* and Their Biological Evaluations

**DOI:** 10.3390/molecules23081920

**Published:** 2018-08-01

**Authors:** Jiwon Baek, Tae Kyoung Lee, Jae-Hyoung Song, Eunyong Choi, Hyun-Jeong Ko, Sanghyun Lee, Sang Un Choi, Seong Lee, Sang-Woo Yoo, Seon-Hee Kim, Ki Hyun Kim

**Affiliations:** 1 School of Pharmacy, Sungkyunkwan University, Suwon 16419, Korea; baekd5nie@gmail.com (J.B.); charmelon8@gmail.com (T.K.L.); 2 College of Pharmacy, Kangwon National University, Chuncheon 24341, Korea; thdwohud@naver.com (J.-H.S.); hjko@kangwon.ac.kr (H.-J.K.); 3 Sungkyun Biotech Co. Ltd., Suwon 16419, Korea; eychoi8812@sungkyunbiotech.co.kr (E.C.); seonhee31@gmail.com (S.-H.K.); 4 Department of Integrative Plant Science, Chung-Ang University, Anseong 17546, Korea; slee@cau.ac.kr; 5 Korea Research Institute of Chemical Technology (KRICT), Daejeon 34114, Korea; suchoi@krict.re.kr; 6 Dankook University Hospital Research Institute of Clinical Medicine, Cheonan 31116, Korea; seonglee@empas.com; 7 Research & Development Center, Natural Way Co., Ltd., Pocheon 11160, Korea; nmrnmr@hanmail.net

**Keywords:** *Lespedeza cuneata*, lignan glycoside, flavonoid glycoside, cytotoxicity, adipocyte and osteoblast differentiation

## Abstract

*Lespedeza cuneata* (Fabaceae), known as Chinese bushclover, has been used in traditional medicines for the treatment of diseases including diabetes, hematuria, and insomnia. As part of a continuing search for bioactive constituents from Korean medicinal plant sources, phytochemical analysis of the aerial portion of *L. cuneata* led to the isolation of two new lignan glycosides (**1**,**2**) along with three known lignan glycosides (**3**–**7**) and nine known flavonoid glycosides (**8**–**14**). Numerous analysis techniques, including 1D and 2D NMR spectroscopy, CD spectroscopy, HR-MS, and chemical reactions, were utilized for structural elucidation of the new compounds (**1**,**2**). The isolated compounds were evaluated for their applicability in medicinal use using cell-based assays. Compounds **1** and **4**–**6** exhibited weak cytotoxicity against four human breast cancer cell lines (Bt549, MCF7, MDA-MB-231, and HCC70) (IC_50_ < 30.0 μM). However, none of the isolated compounds showed significant antiviral activity against PR8, HRV1B, or CVB3. In addition, compound **10** produced fewer lipid droplets in Oil Red O staining of mouse mesenchymal stem cells compared to the untreated negative control without altering the amount of alkaline phosphatase staining.

## 1. Introduction

*Lespedeza cuneata* (Dum. Cours.) G. Don. (Fabaceae), known as Chinese bushclover, is a warm-season, perennial legume that is widely distributed in Korea, China, and India [1]. This plant has been used in folk medicine for the treatment of diseases, including diabetes, hematuria, and insomnia, as well as for the protection of the kidneys, liver, and lungs [2,3]. Previous pharmacological studies of this medicinal plant have revealed that extracts of *L. cuneata* exhibit inhibition of inflammatory mediators in Lipopolysaccharide (LPS)-activated RAW264.7 cells and paw edema in carrageenan-stimulated rats [4], as well as hepatoprotective and antidiabetic effects [1,2,5,6]. A recent study of *L. cuneata* extract reported its in vitro cytotoxic effects against several cancer cell lines including HeLa, Hep3B, A549, and Sarcoma180 [7]. In terms of phytochemical components, it is a rich source of various compounds such as steroids, flavonoids, phenolics [3,6,8], phenylpropanoids [2,9], lignans [5,9], and phenyldilactones [10]. Among the constituents, lignans, and flavonoids are the main components of *L. cuneata*, and the lignans were found to have hepatoprotective [5] and anti-ulcerative colitis activities [9], and the flavonoids were reported to show hepatoprotective [6] and NO-inhibitory effects [11].

**Figure 1:**
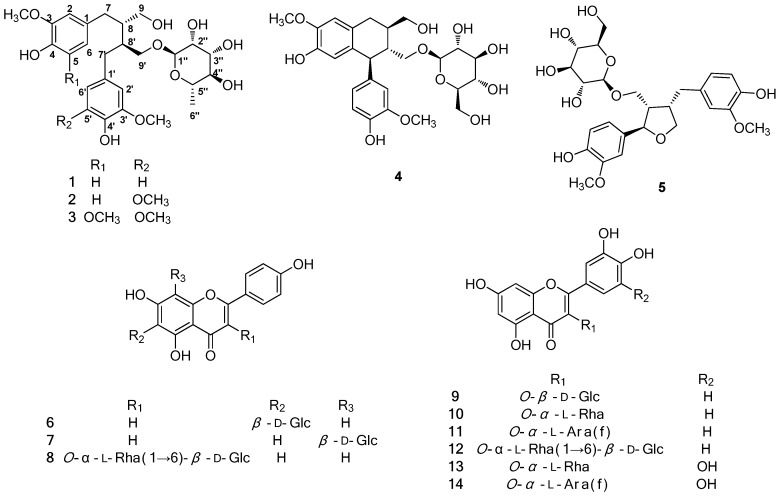
Chemical structures of compounds **1**–**14**. Glc, glucopyranosyl; Rha, rhamnopyranosyl; Ara(f), arabinofuranosyl.

As part of a continuing search for bioactive constituents from Korean medicinal plant sources [12,13,14], the methanol (MeOH) extract of the aerial portion of *L. cuneata* was found to exhibit cytotoxic effects on human ovarian carcinoma cells [15]. In our recent study, bioassay-guided fractionation and repeated chromatography of the MeOH extract of *L. cuneata* resulted in isolation of (−)-9′-*O*-(α-l-rhamnopyranosyl)lyoniresinol, which suppresses the proliferation of A2780 human ovarian carcinoma cells through induction of apoptosis [15]. In the current study investigating bioactive compounds from the aerial portion of *L. cuneata*, further phytochemical analysis was carried out, which led to the isolation of two new lignan glycosides (**1**,**2**) along with three known lignan glycosides (**3**–**7**) and nine known flavonoid glycosides (**8**–**14**). Numerous analysis techniques, including 1D and 2D NMR spectroscopy, CD spectroscopy, HR-MS, and chemical reactions, were utilized for structural elucidation of the new compounds (**1**,**2**). Subsequently, we investigated the possible therapeutic effects of the isolated compounds using various cell-based assays. In this paper, we describe the isolation and structural characterization of compounds **1**–**14** (Figure 1), as well as the evaluation of their applicability to medicinal use including their cytotoxicity, antiviral activity, and their effects on the regulation of adipocyte and osteoblast differentiation. 

## 2. Results and Discussion

### 2.1. Isolation of the Compounds

The dried aerial portion of *L. cuneata* was extracted with 80% MeOH to produce the methanolic extract, which was sequentially solvent-partitioned with hexane, CH_2_Cl_2_, EtOAc, and *n*-BuOH to obtain each solvent fraction. Phytochemical analysis of the EtOAc fraction using repeated column chromatography and high performance liquid chromatography (HPLC) purification led to the isolation of two new lignan glycosides (**1,2**) along with three known lignan glycosides (**3**–**7**) and nine known flavonoid glycosides (**8**–**14**) (Figure 1). 

### 2.2. Structure Elucidation of the Compounds

**Figure 2:**
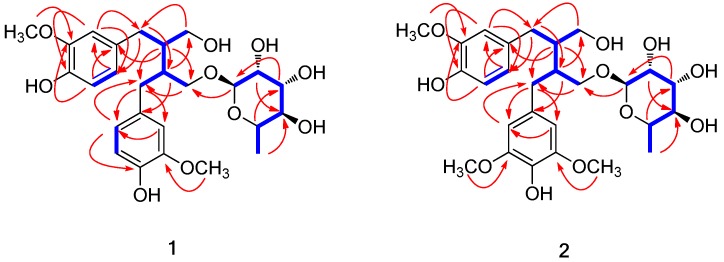
^1^H-^1^H COSY (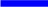) and key HMBC (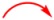) correlations for **1** and **2**.

Compound (**1**) was isolated as a colorless gum with an optical rotation of $({\left[\alpha \right]}_{D}^{25}$ +24.0 (*c* 0.05, MeOH). The molecular formula was determined to be C_26_H_36_O_10_ from the molecular ion peak [M + H]^+^ at *m*/*z* 509.2384 (calculated for C_26_H_37_O_10_ 509.2387) in positive mode High-resolution electrospray ionisation mass spectrometry (HRESIMS) and the NMR spectroscopic data (Table 1). The infrared (IR) spectrum exhibited absorptions of hydroxy groups (3351 cm^−1^) and phenyl rings (1521 and 1455 cm^−1^). The ^1^H NMR spectrum (Table 1) showed signals from two sets of aromatic protons, one at *δ*_H_ 6.67 (1H, d, *J* = 8.0 Hz, H-5), 6.56 (1H, d, *J* = 2.0 Hz, H-2), and 6.53 (1H, dd, *J* = 8.0, 2.0 Hz, H-6) and another at *δ*_H_ 6.66 (1H, d, *J* = 8.0 Hz, H-5’), 6.54 (1H, d, *J* = 2.0 Hz, H-2’), and 6.53 (1H, dd, *J* = 8.0, 2.0 Hz, H-6’), as well as two methoxy groups at *δ*_H_ 3.74 (3H, s) and 3.73 (3H, s). The characteristic NMR data of **1**, combined with heteronuclear single quantum correlation (HSQC) data, also showed signals for four methylenes at δ_H_ 3.77 (1H, dd, *J* = 10.0, 6.0 Hz, H-9’a) and 3.33 (1H, m, H-9’b)/δ_C_ 69.7 (C-9’), δ_H_ 3.69 (1H, m, H-9a), and 3.48 (1H, dd, *J* = 11.0, 7.0 Hz, H-9b)/δ_C_ 62.6 (C-9), δ_H_ 2.67 (1H, dd, *J* = 14.0, 7.0 Hz, H-7a) and 2.56 (1H, dd, *J* = 14.0, 8.5 Hz, H-7b)/δ_C_ 35.6 (C-7), and δ_H_ 2.60 (2H, m, H-7’)/δ_C_ 35.8 (C-7’), and two methines at δ_H_ 2.07 (1H, m, H-8’)/δ_C_ 40.7 (C-8’) and 1.94 (1H, m, H-8)/δ_C_ 44.1 (C-8), which are indicative of a secoisolariciresinol-type lignan [16,17]. In addition, characteristic rhamnose NMR signals were observed at δ_H_ 4.63 (1H, d, *J* = 1.5 Hz, H-1’’) and 1.25 (3H, d, *J* = 6.0 Hz, H-6’’), δ_C_ 102.0, 73.7, 72.4, 72.2, 69.9, and 17.8 [18]. These data suggest that compound **1** is a secoisolariciresinol-type lignan glycoside, and the ^1^H and ^13^C NMR spectra of **1** were highly similar to those of (−)-secoisolariciresinol-*O*-α-l-rhamnopyranoside [19]. The planar gross structure of **1** was established based on the ^1^H-^1^H correlation spectroscopy (COSY) and Heteronuclear multiple bond correlation (HMBC) spectral data (Figure 2). However, the absolute stereochemistry of **1** was not identical to (−)-secoisolariciresinol-*O*-α-l-rhamnopyranoside because compound **1** showed a positive optical rotation (${\left[\alpha \right]}_{D}^{25}$ +24.0, *c* 0.05, MeOH) similar to chaenomiside F (compound **3**) (${\left[\alpha \right]}_{D}^{25}$ +30.0, *c* 0.1, MeOH) [20] and (−)-secoisolariciresinol-*O*-α-l-rhamnopyranoside showed a negative rotation $({\left[\alpha \right]}_{D}^{20}$ −49.5, *c* 0.30, acetone) [19]. Enzymatic hydrolysis was carried out to further confirm the absolute configuration of compound **1**, which yielded an aglycone and a rhamnose. The aglycone was determined to be (+)-secoisolariciresinol (**1a**) through LC/MS analysis with an *m*/*z* signal of 361.2 [M − H]^−^ and a positive optical rotation (${\left[\alpha \right]}_{D}^{25}$ +30.0, *c* 0.02, acetone) [16]. The CD spectrum of **1a** showed positive Cotton effects at 209, 223, and 288 nm, and negative effects at 216 and 230 nm, which is the first report of an experimental CD spectrum of (+)-secoisolariciresinol. The coupling constant (*J* = 1.5 Hz) of the anomeric proton of the rhamnose revealed the α-configuration of the anomeric proton [21]. The identity of l-rhamnose was established through LC/MS analysis of the rhamnose obtained from the enzymatic hydrolysis [22,23]. Thus, the structure of compound **1** was determined to be (+)-secoisolariciresinol-*O*-α-l-rhamnopyranoside. 

Compound **2** was obtained as a colorless gum with a positive optical rotation value of ${\left[\alpha \right]}_{D}^{25}$ +27.5 (*c* 0.04, MeOH). The molecular formula of **2** was determined to be C_27_H_38_O_11_ from the molecular ion peak at *m*/*z* 537.2343 [M − H]^−^ (calculated for C_27_H_37_O_11_ 537.2336) in the negative mode HRESIMS and the NMR spectroscopic data (Table 1). The ultraviolet (UV) and IR spectra of **2** were almost identical to those of **1**. The ^1^H and ^13^C NMR spectra (Table 1) were also quite similar to those of **1**, with a noticeable difference being that the proton signals for a 1,3,4-trisubstituted aromatic ring in **1** were absent and the proton signals for a typical 1,3,4,5-tetrasubstituted aromatic ring (δ_H_ 6.28 (2H, s)) and an overlapped signal for two methoxyl groups (δ_H_ 3.74 (6H, s)) was present in **2**. In light of these data, compound **2** was also deduced to be one of the secoisolariciresinol-type lignans like compound **1**, and the differences in the structure of **2** compared to compound **1** were confirmed through analysis of the ^1^H-^1^H COSY and HMBC data (Figure 2). Specifically, an HMBC correlation from the methoxyl group (δ_H_ 3.74) to C-3’/C-5’ (δ_C_ 147.6) was observed, which led to the assignment of the methoxyl group at C-3’/C-5’. The similarity between the characteristic CD curves of **1** (positive at 206, 229, and 285 nm and negative at 217 nm) and **2** (positive at 205, 233, and 283 nm and negative at 221 nm) revealed that the absolute configuration of **2** was identical to compound **1** as the 8S and 8’S form, which was also supported by the positive optical rotation value (${\left[\alpha \right]}_{D}^{25}$ +27.5, *c* 0.04, MeOH) of **2** like that of **1**. Enzymatic hydrolysis was conducted to further confirm the absolute configuration of **2**, which yielded an aglycone (**2a**) and a rhamnose. As expected, the aglycone (**2a**) was determined to be (+)-seco-5’-methoxy-isolariciresinol using LC/MS analysis with an *m*/*z* signal of 393.2 [M + H]^+^ and a positive optical rotation value of **2a** (${\left[\alpha \right]}_{D}^{25}$ +25.5, *c* 0.02, acetone) [16]. The characteristic small coupling constant (*J* = 1.5 Hz) of the anomeric proton of the rhamnose at δ_H_ 4.64 indicated the α-configuration of the rhamnose [21], and l-rhamnose was confirmed using LC/MS analysis of the rhamnose obtained from the enzymatic hydrolysis of **2** [22,23]. Accordingly, the structure of compound **2** was determined to be (+)-seco-5’-methoxy-isolariciresinol-9’-*O*-α-l-rhamnopyranoside.

The known compounds were identified as chaenomiside F (**3**) [16,20], (+)-isolariciresinol 9-*O*-β-d-glucoside (**4**) [5], lariciresinol 9-*O*-β-d-glucopyranoside (**5**) [24], isovitexin (**6**) [25], vitexin (**7**) [26], nicotiflorin (**8**) [27], isoquercetin (**9**) [28], quercimelin (**10**) [29], avicularin (**11**) [30], rutin (**12**) [28], myricitrin (**13**) [31], and betmidin (**14**) [32,33], through comparison of their spectroscopic data, including ^1^H and ^13^C NMR, and physical data with previously reported values, as well as through LC/MS analysis.

### 2.3. Cytotoxic Activity of Isolated Compounds against Human Tumor Cell Lines

Based on the cytotoxic activity of the MeOH extract of *L. cuneata* in our recent study [15], the cytotoxic activities of the isolated compounds (**1**–**14**) were evaluated by determining their inhibitory effects on human tumor cell growth in human breast cancer cells (Bt549, MCF7, MDA-MB-231 and HCC70), using a sulforhodamine B (SRB) bioassay [12,34]. The results (Table S1) demonstrated that compound **1** showed cytotoxicity against Bt549, MDA-MB-231, and HCC70 cell lines with IC_50_ values ranging from 24.38–26.16 μM. Compounds **4** and **5** exhibited cytotoxicity against MCF7 (IC_50_: 28.08 μM) and HCC70 (IC_50_: 24.81 μM) cell lines, respectively, and compound **6** showed cytotoxic activity against MCF7, MDA-MB-231, and HCC70 cell lines with IC_50_ values ranging from 27.57–29.18 μM (Table S1). However, other compounds were inactive (IC_50_ > 30.0 μM). Although recent studies of *L. cuneata* extract have reported that the extract showed cytotoxic effects against various cancer cell lines [7,15], the isolated compounds (**1**–**14**) did not appear to be responsible for the cytotoxicity. 

### 2.4. Antiviral Activity of the Isolated Compounds against PR8, HRV1B, and CVB3 Infection

Recently, many studies exploring antiviral natural products and organic synthetic compounds have reported that a variety of flavonoids exhibit potent antiviral activity by inhibiting the early stages of viral infection, viral protein expression, and neuraminidase activity [35,36,37]. Therefore, we assessed the isolated compounds (**1**–**14**) for their antiviral activity against PR8, HRV1B, and CVB3 infection in A549, Vero, and HeLa cells, respectively. Less than 10% of the cells survived in the positive-control group (cells with virus only) after 48 hours of infection. In addition, cells treated with compounds **1**–**14** (10 μM) also had less than 10% survival. Because we could not identify any significant differences between the control and test groups, these results suggest that the compounds do not show significant antiviral activity against PR8, HRV1B, or CVB3.

### 2.5. Regulatory Effects of Compound ***10*** on Differentiation into Adipocytes and Osteoblasts

**Figure 3:**
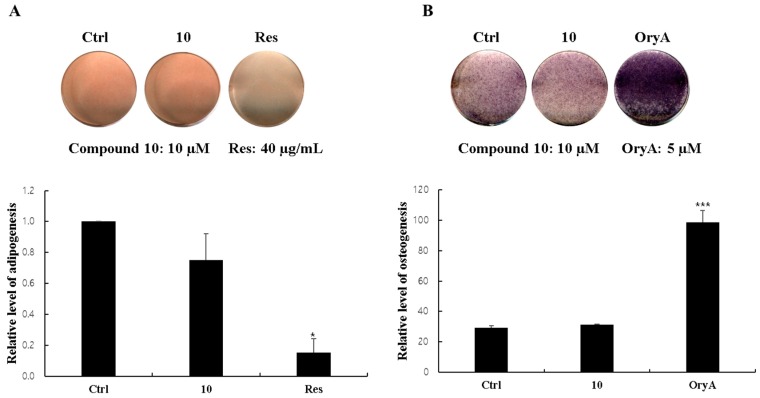
Reciprocal effects of compound **10** on the differentiation of MSCs into adipocytes or osteoblasts. Mouse mesenchymal stem cells (C3H10T1/2) were treated with 10 μM compound **10***.* After adipogenic differentiation, the cells were stained with Oil Red O (ORO), and the number of stained lipid droplets was quantitatively evaluated (**A**). After osteoblast differentiation, the cells were stained for ALP levels, and the ALP activity was measured (**B**). Ctrl represents untreated negative control. For the positive controls, 40 micrograms of resveratrol (Res) was used for adipogenesis and 5 μM of oryzativol A (OryA) was added for osteogenesis. * denotes 0.01 ≤ *p* ≤ 0.05 and *** denotes *p* ˂ 0.001.

Mesenchymal stem cells (MSCs) in the bone marrow are pluripotent cells, which differentiate into osteocytes as well as adipocytes. Since microenvironmental changes such as hormones, immune responses, and metabolism cause alterations in the regulation of MSC differentiation, where alterations in the expression of the related genes might disturb the balance between osteoprogenitor and adipocyte progenitor cells in osteoporosis patients [38], natural products that are able to suppress MSC differentiation toward adipocytes and/or promote osteogenic differentiation of MSC would be promising in the management of postmenopausal osteoporosis. The biological activity of compound **10** was additionally tested regarding its effects on the differentiation of mouse MSCs into adipocytes or osteoblasts, since large amounts of compound **10** was isolated among the isolated compounds. Compound **10** was added to the MSC culture media during adipocyte differentiation. Compound **10** slightly reduced the formation of lipid droplets and resulted in somewhat fewer Oil Red O (ORO)-stained cells compared to the normally differentiated adipocytes (Figure 3A). However, ALP staining and ALP activity in the compound **10**-treated cells did not increase during the MSC differentiation into osteoblasts, in contrast to the positive control group treated with oryzativol A (Figure 3B). These results demonstrate that compound **10** marginally suppressed adipogenesis of MSCs but did not influence osteogenesis. 

## 3. Materials and Methods 

### 3.1. Plant Material

The aerial portions of *L. cuneata* were collected from Mt. Bangtae, Inje, Kangwon Province, Republic of Korea, in October 2016. The plant materials were identified by one of the authors, Prof. S. Lee. A voucher specimen (YKM-2016) was deposited at the herbarium of the School of Pharmacy, Sungkyunkwan University, Suwon, Republic of Korea.

### 3.2. Extraction and Isolation

The dried aerial portions of *L. cuneata* (4.2 kg) were extracted three times with 4.2 L of 80% MeOH for three days at room temperature and filtered. The resultant filtrate was evaporated under reduced pressure using a rotavap to obtain the MeOH extract (401.8 g), which was suspended in distilled H_2_O (2 L) and successively solvent-partitioned with hexane, CH_2_Cl_2_, EtOAc, and *n*-BuOH (2.0 L × 3 for each) to yield the hexane- (20.6 g), CH_2_Cl_2_- (0.7 g), EtOAc- (12.7 g), and *n*-BuOH-soluble (69.3 g) fractions. The EtOAc-soluble fraction (12.7 g) was subjected to Diaion HP-20 column chromatography with a gradient solvent system of MeOH-H_2_O (0–100% MeOH) to afford six fractions (A–F). Fraction D (5.4 g) was separated using RP-C18 column chromatography with a gradient solvent system of MeOH-H_2_O (30–100% MeOH) to yield six sub-fractions (D_1_–D_6_). Sub-fraction D_3_ (2.8 g) was fractionated using silica gel column chromatography with a gradient solvent system of CH_2_Cl_2_-MeOH-H_2_O (15:1:0–9:3:0.5 *v*/*v*/*v*) to produce 10 sub-fractions (D_3_-1–D_3_-10). Sub-fraction D_3_-7 (1.1 g) was separated using an RP-C18 column with 60% MeOH to produce four sub-fractions (D_3_-71–D_3_-74). Sub-fraction D_3_-72 (506.7 mg) was subjected to silica gel column chromatography with a gradient solvent system of CH_2_Cl_2_-MeOH-H_2_O (10:1:0–1:1:0.25, *v*/*v*/*v*) to give five sub-fractions (D_3_-721–D_3_-725). Sub-fraction D_3_-722 (316.4 mg) was subjected to Sephadex LH-20 column chromatography with 100% MeOH to produce 10 sub-fractions (D_3_-722A–D_3_-722J). Sub-fraction D_3_-722C (230.0 mg) was purified using semi-preparative HPLC with a Phenomenex Luna phenyl-hexyl column (18% MeCN, flow rate: 2 mL/min) to yield compound **5** (1.4 mg, *t_R_* = 37.0 min). Sub-fraction D_3_-73 (158.8 mg) was subjected to Sephadex LH-20 column chromatography with 100% MeOH to give 10 sub-fractions (D_3_-73A–D_3_-73J). Compounds **2** (0.7 mg, *t_R_* = 49.5 min) and **3** (1.8 mg, *t_R_* = 41.5 min) were obtained from sub-fraction D_3_-73B (24.5 mg) using semi-preparative HPLC with a Phenomenex Luna phenyl-hexyl column (18% MeCN, flow rate: 2 mL/min). Compound **1** (7.6 mg, *t_R_* = 61.0 min) was isolated from sub-fraction D_3_-73C (44.7 mg) using semi-preparative HPLC with a Phenomenex Luna phenyl-hexyl column (18% MeCN, flow rate: 2 mL/min). Compound **14** (3.7 mg, *t_R_* = 20.5 min) was obtained from sub-fraction D_3_-73I (8.2 mg) using semi-preparative HPLC with a Phenomenex Luna phenyl-hexyl column (21% MeCN, flow rate: 2 mL/min). Sub-fraction D_3_-74 (127.6 mg) was subjected to Sephadex LH-20 column chromatography with 100% MeOH to give eight sub-fractions (D_3_-741–D_3_-748). Compounds **9** (0.7 mg, *t_R_* = 30.5 min) and **10** (32.8 mg, *t_R_* = 48.0 min) were isolated from sub-fraction D_3_-746 (42.3 mg) using semi-preparative HPLC with a Phenomenex Luna phenyl-hexyl column (18% MeCN, flow rate: 2 mL/min). Sub-fraction D_3_-8 (515.0 mg) was subjected to RP-C18 column chromatography using a gradient solvent system of 40–60% MeOH to produce four sub-fractions (D_3_-81–D_3_-84). Sub-fraction D_3_-82 (346.7 mg) was subjected to silica gel column chromatography with a gradient solvent system of CH_2_Cl_2_-MeOH (10:1–1:1, *v*/*v*) to give four sub-fractions (D_3_-821–D_3_-824). Sub-fraction D_3_-822 (54.8 mg) was applied to Sephadex LH-20 column chromatography with 100% MeOH to produce six sub-fractions (D_3_-822A–D_3_-822F). Compound **4** (3.5 mg, *t_R_* = 39.0 min) was purified from sub-fraction D_3_-822A (16.3 mg) using semi-preparative HPLC with a Phenomenex Luna phenyl-hexyl column (15% MeCN, flow rate: 2 mL/min). Sub-fraction D_3_-824 (78.1 mg) was separated using Sephadex LH-20 column chromatography with 100% MeOH to yield five sub-fractions (D_3_-824A–D_3_-824E). Sub-fraction D_3_-824C (22.4 mg) was separated using semi-preparative HPLC with a Phenomenex Luna phenyl-hexyl column (16% MeCN, flow rate: 2 mL/min) to obtain compound **8** (2.3 mg, *t_R_* = 72.5 min). Sub-fraction D_3_-824D (37.3 mg) was separated using semi-preparative HPLC with a Phenomenex Luna phenyl-hexyl column (14% MeCN, flow rate: 2 mL/min) to obtain compound **13** (0.5 mg, *t_R_* = 73.0 min), and compound **13**’s washing fraction D_3_-824DW (20.5 mg) was collected. Compound **11** (1.0 mg, *t_R_* = 49.5 min) was purified using semi-preparative HPLC with a Phenomenex Luna phenyl-hexyl column (18% MeCN, flow rate: 2 mL/min) from sub-fraction D_3_-824DW (20.5 mg). Sub-fraction D_3_-10 (132.7 mg) was applied to Sephadex LH-20 column chromatography with 80% MeOH to produce nine sub-fractions (D_3_-101–D_3_-109). Sub-fraction D_3_-108 (50.3 mg) was further separated using semi-preparative HPLC with a Phenomenex Luna phenyl-hexyl column (38% MeOH, flow rate: 2 mL/min) to yield compound **12** (2.1 mg, *t_R_* = 72.0 min). Finally, compounds **6** (0.6 mg, *t_R_* = 37.0 min) and **7** (2.0 mg, *t_R_* = 39.0 min) were isolated from sub-fraction D_3_-109 (17.2 mg) using semi-preparative HPLC with a Phenomenex Luna phenyl-hexyl column (20% MeCN, flow rate: 2 mL/min).

#### 3.2.1. (+)-Secoisolariciresinol-*O*-α-l-rhamnopyranoside (**1**)

Colorless gum; ${\left[\alpha \right]}_{D}^{25}$ +24.0 (*c* = 0.05, MeOH); ESIMS (negative mode) *m*/*z:* 507 [M − H]^−^; HRESIMS (positive mode) *m*/*z:* 509.2384 [M + H]^+^, calculated for C_26_H_37_O_10_, 509.2387; UV (MeOH) λ_max_ nm (log ε): 205 (2.29), 233 (3.43), 283 (0.76); IR (KBr) ν_max_ cm^−1^: 3703, 3351, 2947, 2833, 2513, 2302, 2047, 1521, 1455; CD (MeOH) λ_max_ nm (Δε): 206 (+19.2), 217 (−11.5), 229 (+10.3), 285 (+2.8); ^1^H (CD_3_OD, 800 MHz) and ^13^C (CD_3_OD, 200 MHz) NMR spectroscopic data, see Table 1.

#### 3.2.2. (+)-Seco-5’-methoxy-isolariciresinol-9’-*O*-α-l-rhamnopyranoside (**2**)

Colorless gum; ${\left[\alpha \right]}_{D}^{25}$ +27.5 (*c* = 0.04, MeOH); ESIMS (negative mode) *m*/*z:* 537 [M − H]^−^; HRESIMS (negative mode) *m*/*z:* 537.2343 [M − H]^−^, calculated for C_27_H_37_O_11_, 537.2341; UV (MeOH) λ_max_ nm (log ε): 205 (2.29), 233 (3.43), 283 (0.76); IR (KBr) ν_max_ cm^−1^: 3705, 3340, 2945, 2831, 2512, 2302, 2045, 1516, 1453; CD (MeOH) λ_max_ nm (Δε): 205 (+11.5), 221 (−23.4), 233 (+13.8), 283 (+3.1); ^1^H (CD_3_OD, 800 MHz) and ^13^C (CD_3_OD, 200 MHz) NMR spectroscopic data, see Table 1.

### 3.3. Enzymatic Hydrolysis of Compounds ***1***,***2***

A solution of each compound (1.0 mg) in H_2_O (1 mL) was individually hydrolyzed with naringinase (10 mg, from *Penicillium* sp.; ICN Biomedicals Inc., Irvine, CA, USA) at 40 °C for 36 h. Each reaction mixture was extracted with CH_2_Cl_2_ to yield the individual CH_2_Cl_2_ extract and a water phase. The CH_2_Cl_2_ extracts from compounds **1** and **2** were chromatographically separately with a Phenomenex Strata^®^ C18-E column (2 g) using a gradient solvent system from 100% H_2_O to 100% MeOH to give aglycones **1a** (0.3 mg) and **2a** (0.3 mg), respectively. The aglycone of **1a** was determined to be (+)-secoisolariciresinol using LC/MS analysis with an *m*/*z* signal of 361.2 [M − H]^−^ and a positive optical rotation (${\left[\alpha \right]}_{D}^{25}$ +30.0, *c* 0.02, acetone) [16]. The CD spectrum of **1a** showed positive Cotton effects at 209, 223, and 288 nm and negative effects at 216 and 230 nm. The aglycone of **2a** was determined to be (+)-seco-5’-methoxy-isolariciresinol using LC/MS analysis with an *m*/*z* signal of 393.2 [M + H]^+^ and a positive optical rotation (${\left[\alpha \right]}_{D}^{25}$ +25.5, *c* 0.02, acetone) [16]. After drying the water phase in vacuo, the residue was dissolved in anhydrous pyridine (200 μL) followed by the addition of l-cysteine methyl ester hydrochloride (0.6 mg). The reaction mixture was incubated at 60 °C for 1 h, then *O*-tolyl isothiocyanate (15 μL) was added and the mixture was incubated at 60 °C for 1 h. The reaction product was directly analyzed using LC/MS (0−35% MeCN for 30 min, flow rate: 0.3 mL/min) with an analytical Kinetex column (2.1 × 100 mm, 5 μm) (Agilent Technologies, Santa Clara, CA, USA). The l-rhamnose in compounds **1** and **2** was identified through comparison of the retention times with those of authentic sample (*t_R_* = l-rhamnose 25.6 min).

### 3.4. Cytotoxicity Assay

A sulforhodamine B (SRB) bioassay was used to determine the cytotoxicity of each isolated compound against four cultured human tumor cell lines [12,34]. The assays were performed at the Korea Research Institute of Chemical Technology. All the cell lines used, Bt549, MCF7, MDA-MB-231, and HCC70, are human breast cancer cells. Etoposide (purity ≥ 98%, Sigma, St. Louis, MO, USA) was used as a positive control. The half maximal inhibitory concentrations (IC_50_) of cancer cell growth are expressed as the mean from three distinct experiments.

### 3.5. Antiviral Activity Assay

Influenza A/PR/8 virus (PR8), human rhinovirus 1B (HRV1B), and coxsackievirus B3 (CVB3) were purchased from ATCC (American Type Culture Collection, Manassas, VA, USA). PR8, CVB3, and HRV1B were replicated in A549, Vero, and HeLa cells, respectively, at 37 °C. Antiviral activity was evaluated with the SRB method using cytopathic effect (CPE) reduction as previously reported [39].

### 3.6. Oil Red OStaining

At 6–8 days after differentiation, the adipocytes were fixed with 10% neutral buffered formalin (NBF) and stained with 0.5% Oil Red O (Sigma, St. Louis, MO, USA). To stop the reaction, cells were washed with distilled water three times. Stained cells were resolved with 1 mL of isopropanol and the colorimetric changes was measured at 520 nm to evaluate intra-cellular triglyceride content.

### 3.7. Alkaline Phosphatase (ALP) Staining and Activity

At 7–9 days after osteogenic differentiation, the medium was removed, and the cells were washed with 2 mM MgCl_2_ solution. After incubation with AP buffer (100 mM Tris−HCl, pH 9.5, 100 mM NaCl, and 10 mM MgCl_2_) for 15 min, the cells were treated in AP buffer containing 0.4 mg/mL of nitro-blue tetrazolium (NBT, Sigma) and 0.2 mg/mL of 5-bromo-4-chloro-3-indolyl phosphate (BCIP, Sigma) for 15 more minutes. To stop the reaction, the cells were exposed to 5 mM EDTA (pH 8.0) and fixed with 10% NBF for 1 h. 

The differentiation into osteoblast was evaluated regarding ALP activity. The ALP activity was determined using an Alkaline Phosphatase Assay Kit (ab83369; Abcam, Cambridge, MA, USA). Briefly, the cell lysates were incubated with *p*-nitrophenyl phosphate (*p*-NPP) solution at RT for 1 h in the dark. After stopping the reaction, the optical density was measured at 405 nm using a SpectraMax M2/M2e Microplate Readers (Molecular Devices, San Jose, CA, USA). 

## 4. Conclusions

In the present study, phytochemical analysis of the aerial portion of *L. cuneata* led to the isolation of two new lignan glycosides (**1**,**2**) along with three known lignan glycosides (**3**–**7**) and nine known flavonoid glycosides (**8**–**14**). All the isolated compounds were evaluated for their applicability for medicinal use using cell-based assays. Compounds **1** and **4**–**6** exhibited weak cytotoxicity against the breast cancer cell lines (Bt549, MCF7, MDA-MB-231 and HCC70) (IC_50_ < 30.0 μM), while none of the isolated compound showed significant antiviral activity against PR8, HRV1B, or CVB3. In a mouse mesenchymal stem cell line, treatment with compound **10** resulted in fewer lipid droplets compared to the untreated negative without altering the amount of alkaline phosphatase staining.

## Figures and Tables

**Table 1:** ^1^H and ^13^C NMR data of **1** and **2** in CD_3_OD (δ in ppm, 800 MHz for ^1^H and 200 MHz for ^13^C) ^a^.

Position	1			2		
	δ_H_	δ_C_		δ_H_	δ_C_	
1		133.6	s		132.2	s
2	6.56 d (2.0)	113.0	d	6.54 d (2.0)	111.9	d
3	6.67 ^α^ d (8.0)	115.5	d	6.65 d (8.0)	114.2	d
4		145.4	s		144.5	s
5		148.9	s		147.5	s
6	6.53 dd (8.0, 2.0)	122.6	d	6.52 dd (8.0, 2.0)	121.3	d
7	2.67 dd (14.0, 7.0); 2.56 dd (14.0, 8.5)	35.6	t	2.69 dd (14.0, 6.5); 2.53 dd (14.0, 9.0)	34.5	t
8	1.94 m	44.1	d	1.92 m	42.5	d
9	3.69 m; 3.48 dd (11.0, 7.0)	62.6	t	3.71 m; 3.48 dd (11.0, 7.0)	61.2	t
1’		133.6	s		131.4	s
2’	6.54 d (2.0)	113.0	d	6.28 s	105.3	d
3’		148.8	s		147.6	s
4’		145.4	s		133.4	s
5’	6.66 ^α^ d (8.0)	115.5	d		147.6	s
6’	6.53 dd (8.0, 2.0)	122.6	d	6.28 s	105.3	d
7’	2.60 m	35.8	t	2.60 m	35.2	t
8’	2.07 m	40.7	d	2.08 m	39.3	d
9’	3.77 dd (10.0, 6.0); 3.33 m	69.7	t	3.79 dd (10.0, 6.0); 3.35 m	67.9	t
1’’	4.63 d (1.5)	102.0	d	4.64 d (1.5)	100.7	d
2’’	3.82 dd (3.5, 1.5)	72.2	d	3.81 dd (3.5, 1.5)	71.0	d
3’’	3.68 dd (9.5, 3.5)	72.4	d	3.68 dd (9.5, 3.5)	71.1	d
4’’	3.38 t (9.5)	73.7	d	3.38 t (9.5)	72.5	d
5’’	3.62 dq (9.5, 6.0)	69.9	d	3.62 dq (9.5, 6.0)	68.7	d
6’’	1.25 d (6.0)	17.8	q	1.25 d (6.0)	16.5	q
3-OCH_3_	3.73 ^β^ s	55.8	q	3.72 s	54.7	q
3’-OCH_3_	3.74 ^β^ s	55.8	q	3.74 s	55.1	q
5’-OCH_3_				3.74 s	55.1	q

^a^*J* values are in parentheses and reported in Hz; ^13^C NMR assignments based on ^1^H-^1^H COSY, HSQC, and HMBC experiments; ^α, β^ Exchangeable peaks.

## Supplementary Materials

Supplementary materials are available online. General experimental procedures, 1D NMR, 2D NMR, HRESIMS, CD data of **1** and **2**, LC/MS analysis of **1** and **2**, and Table S1 are available free of charge on the Internet.
