# Supplementary material for: Lignan Glycosides and Flavonoid Glycosides from the Aerial Portion of Lespedeza cuneata and Their Biological Evaluations
Source: Molecules. 2018 Aug 1;23(8):1920. doi: 10.3390/molecules23081920 (PMC6222362; doi:10.3390/molecules23081920)
Supplement: Supplementary file 1 [file molecules-23-01920-s001.pdf]

Supplementary Material

Article

Lignan Glycosides and Flavonoid Glycosides from the Aerial Portion of *Lespedeza cuneata* and Their Biological Evaluations

Jiwon Baek [1], Tae Kyoung Lee [1], Jae-Hyoung Song [2], Enuyong Choi [3], Hyun-Jeong Ko [2], Sanghyun Lee [4], Sang Un Choi [5], Seong Lee [6], Sang-Woo Yoo [7], Seon-Hee Kim [3] and Ki Hyun Kim [1,*]

[1] School of Pharmacy, Sungkyunkwan University, Suwon 16419, Korea; baekd5nie@gmail.com (J.B.); charmelon8@gmail.com (T.K.L.)
[2] College of Pharmacy, Kangwon National University, Chuncheon 24341, Korea; thdwohud@naver.com (J.H.S.); hjko@kangwon.ac.kr (H.J.K.)
[3] Sungkyun Biotech Co. Ltd., Suwon 16419, Korea; eychoi8812@sungkyunbiotech.co.kr (E.C.); seonhee31@gmail.com (S.H.K.)
[4] Department of Integrative Plant Science, Chung-Ang University, Anseong 17546, Korea; slee@cau.ac.kr
[5] Korea Research Institute of Chemical Technology (KRICT), Daejeon 34114, Korea; suchoi@krict.re.kr
[6] Dankook University Hospital Research Institute of Clinical Medicine, Cheonan 31116, Korea; seonglee@empas.com
[7] Research & Development Center, Natural Way Co., Ltd., Pocheon 11160, Korea; nmrnmr@hanmail.net
* Correspondence: khkim83@skku.edu; Tel.: +82-31-290-7700

Received: 9 July 2018; Accepted: 30 July 2018; Published: 1 August 2018

General Experimental Procedures

HR-ESI mass spectra were acquired using a Waters Xevo G2 QTOF mass spectrometer with an analytical Acquity UPLC column (2.1 × 100 mm, 1.7 μm) (Waters MS Technologies, Manchester, UK). Optical rotations were measured (path length of the measuring tube, 10 cm) using a Jasco P-1020 polarimeter (Jasco, Easton, MD, USA) at room temperature. Ultraviolet (UV) spectra were acquired using an Agilent 8453 UV-visible spectrophotometer (Agilent Technologies, Santa Clara, CA, USA). IR spectra were acquired using a Bruker IFS–66/S FT-IR spectrometer, and NMR spectra were acquired using a Bruker Avance III HD 800 NMR spectrometer with a 5-mm TCI Cryoprobe (Bruker, Karlsruhe, Germany). Preparative HPLC and semi-preparative HPLC were performed using a Waters 1525 binary HPLC pump with a Waters 996 photodiode array detector and an analytical Agilent Eclipse XDB-C18 column (250 × 21.2 mm, 7 μm) (Waters Corporation, Milford, CT, USA), and a Phenomenex Luna phenyl-hexyl 100 Å column (250 × 10 mm, 10 μm), respectively. LC/MS analysis was performed using an Agilent 1200 Series HPLC system equipped with a diode array detector coupled with a 6130 Series ESI mass spectrometer using an analytical Kinetex column (2.1 × 100 mm, 5 μm) (Agilent Technologies, Santa Clara, CA, USA). Column chromatography was performed with either silica gel 60 (Merck, 230–400 mesh) or RP-C18 silica gel (Merck, 230–400 mesh). Sephadex LH-20 (Pharmacia, Uppsala, Sweden) was used for molecular sieve column chromatography, and Diaion HP-20 (Mitsubishi Chemical, Tokyo, Japan) was used for open-column chromatography. Analytical grade solvents (Samchun Pure Chemicals Co., Ltd., Pyeongtaek, Korea) were used for all fractionation and isolation. Merck precoated silica gel F_{254} plates and reversed-phase (RP)-18 F_{254s} plates were used for TLC, and the spots were detected either under UV light (dual wavelength 254/365 nm) or by heating after spraying with anisaldehyde-sulfuric acid.

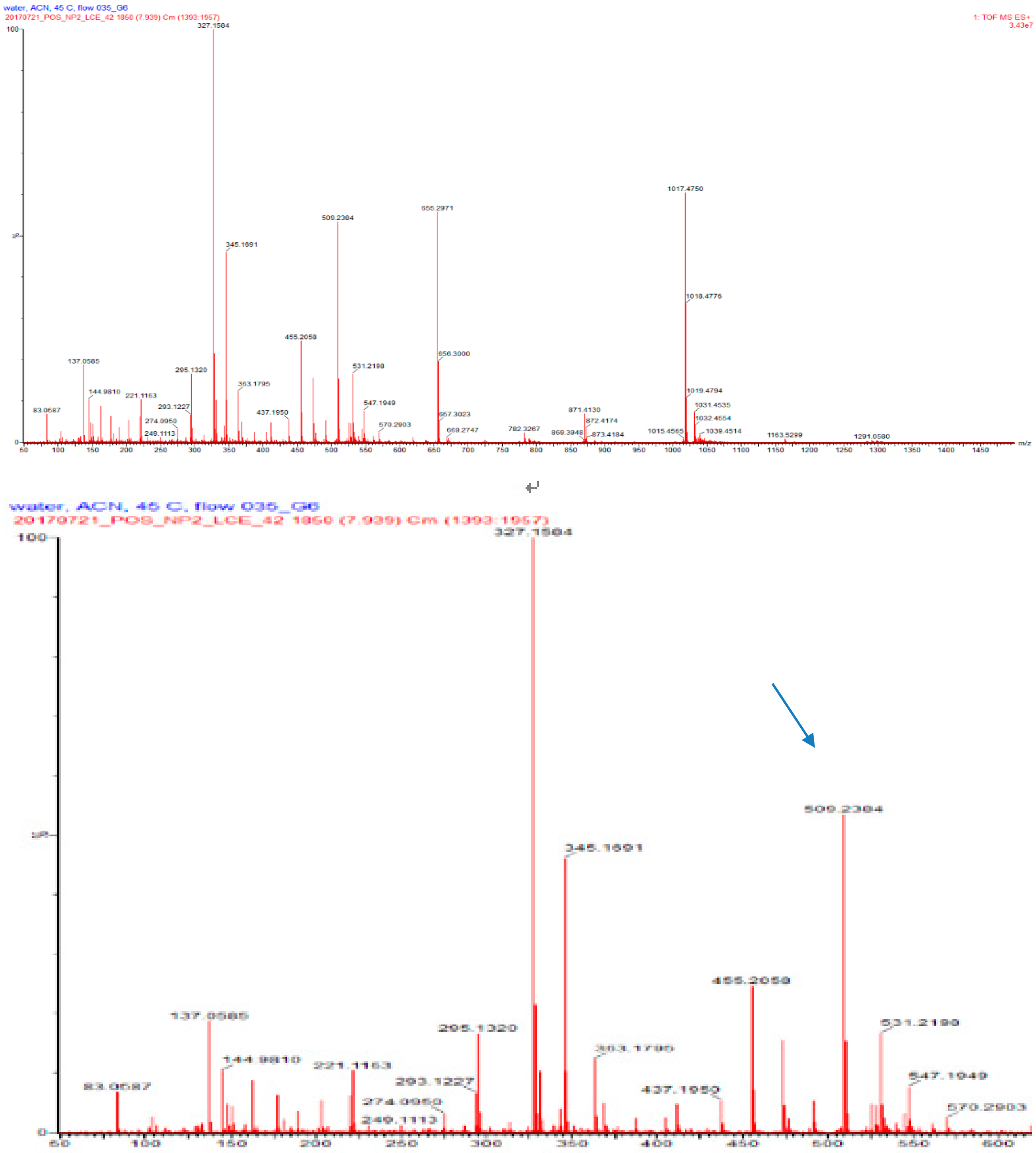

Figure S1. HR-ESI-MS of compound **1.**

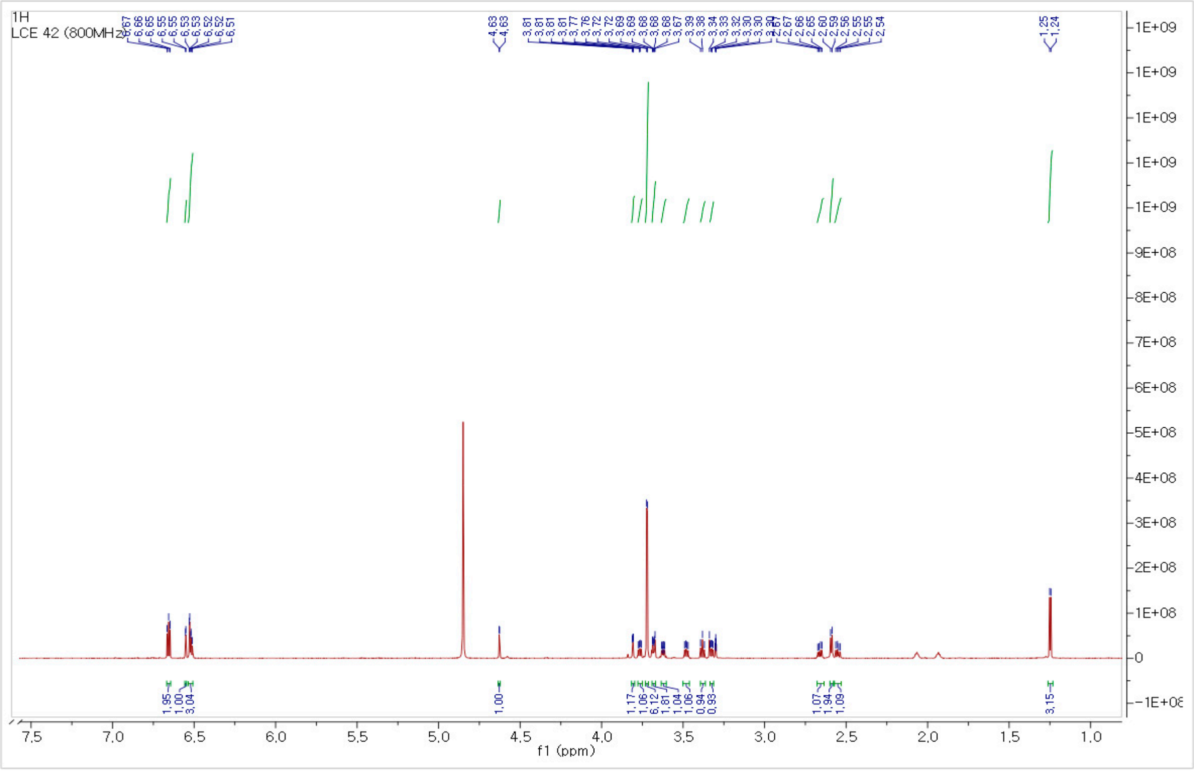

Figure S2. ^{1}H NMR spectrum of compound **1.**

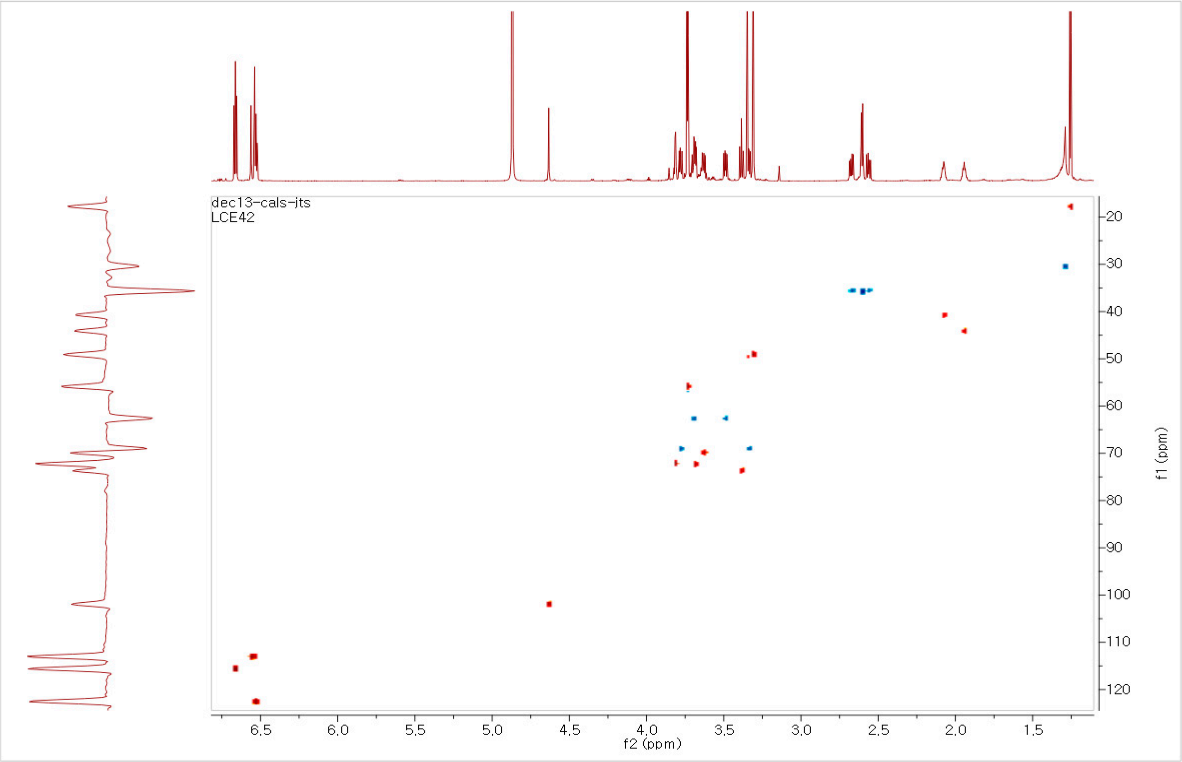

Figure S3. HSQC spectrum of compound **1.**

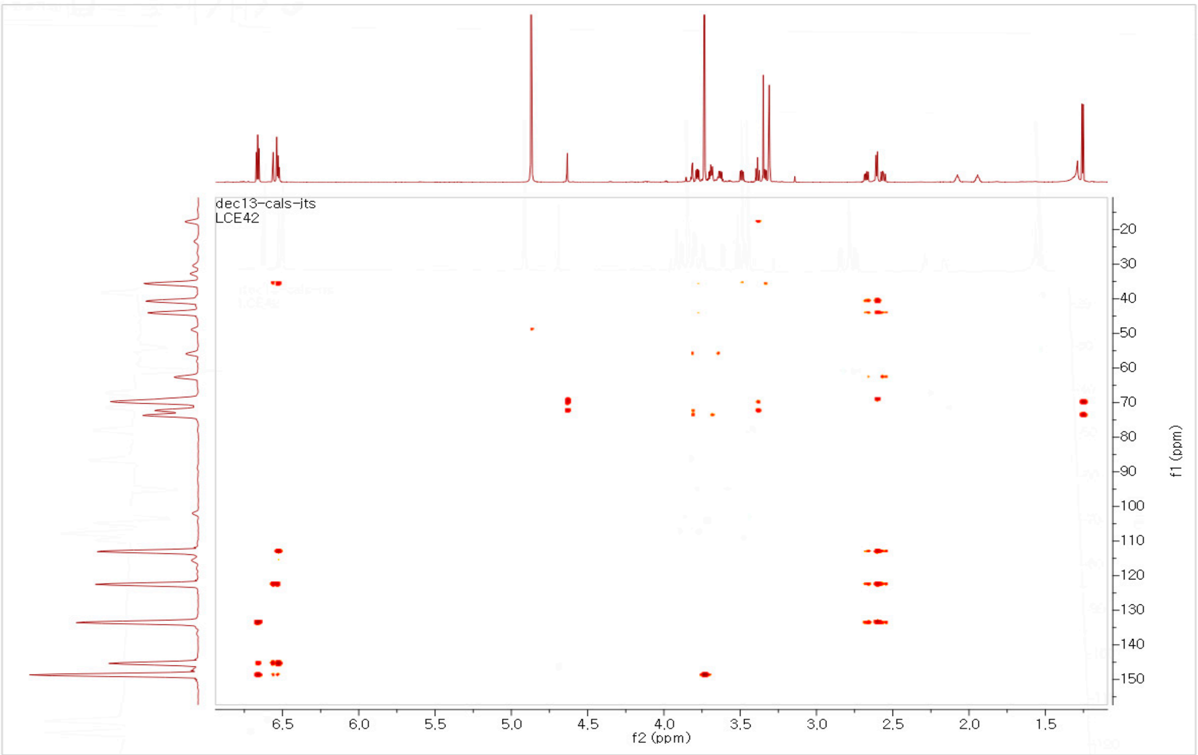

Figure S4. HMBC spectrum of compound **1.**

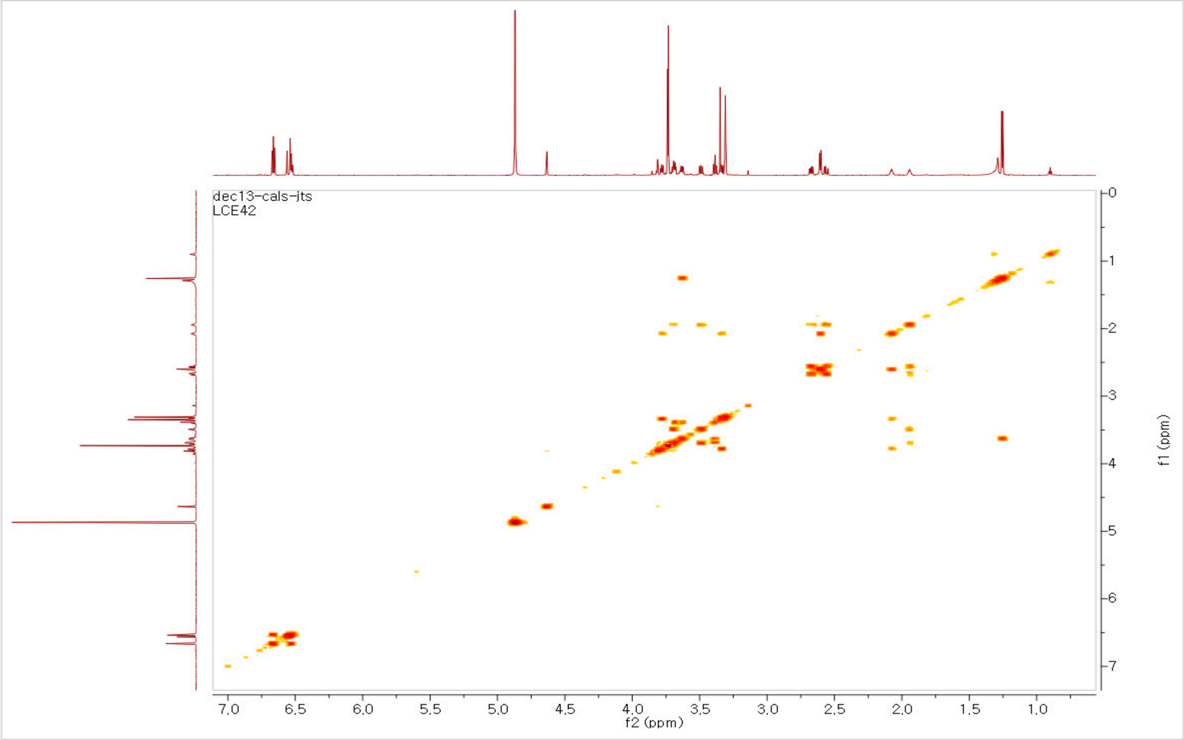

Figure S5. ^{1}H-^{1}H COSY spectrum of compound **1.**

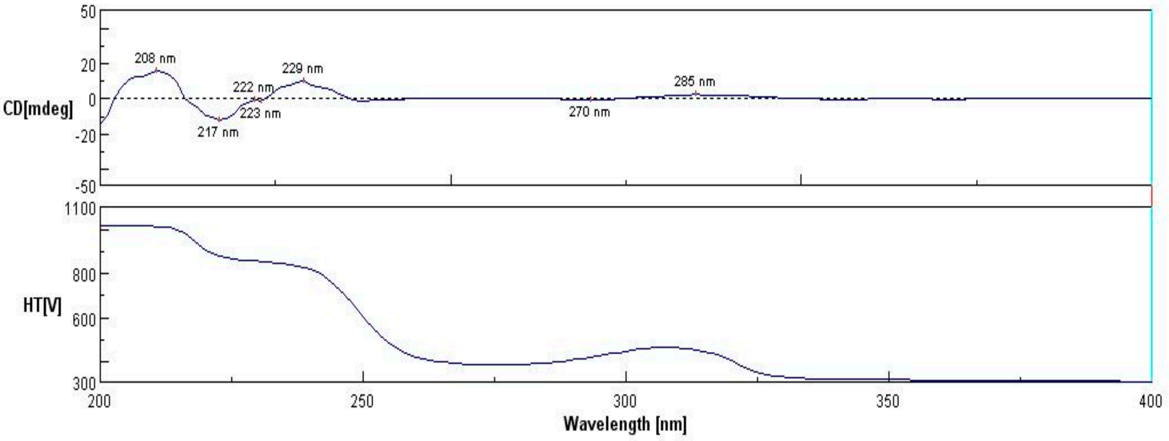

Figure S6. CD spectrum of compound **1.**

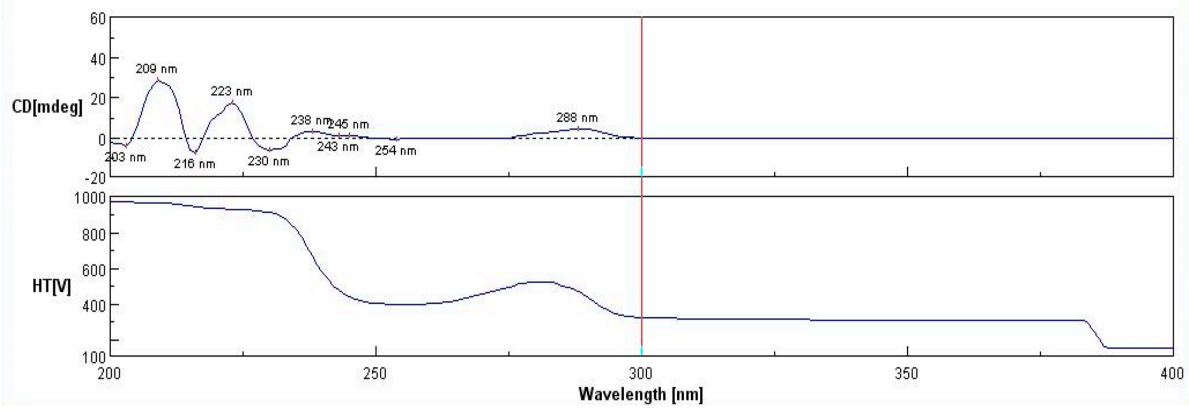

Figure S7. CD spectrum of compound **1a.**

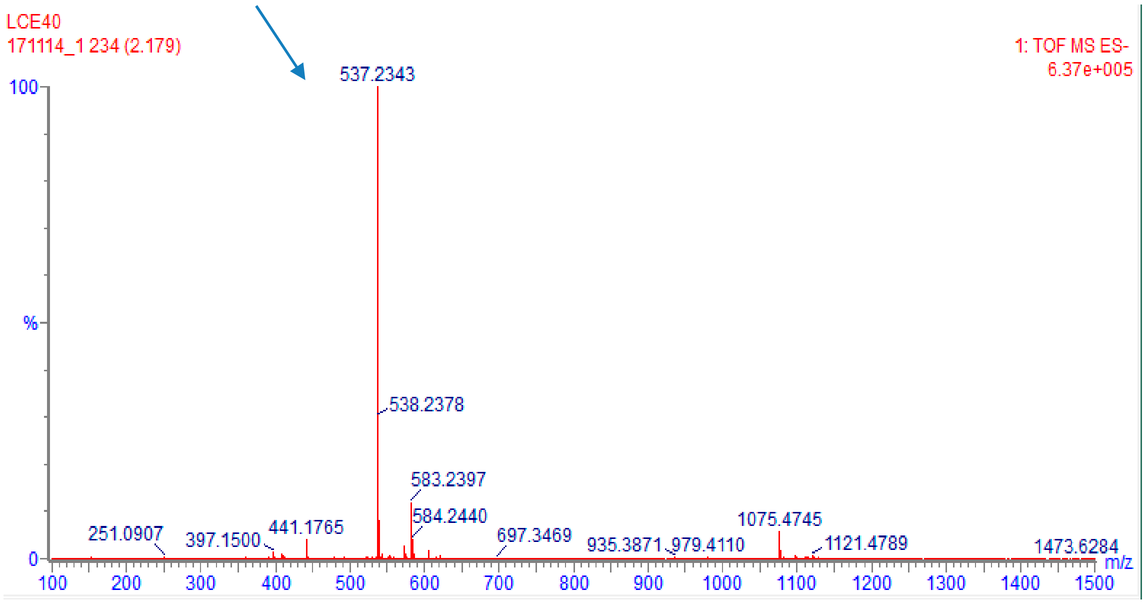

Figure S8. HR-ESI-MS of compound **2.**

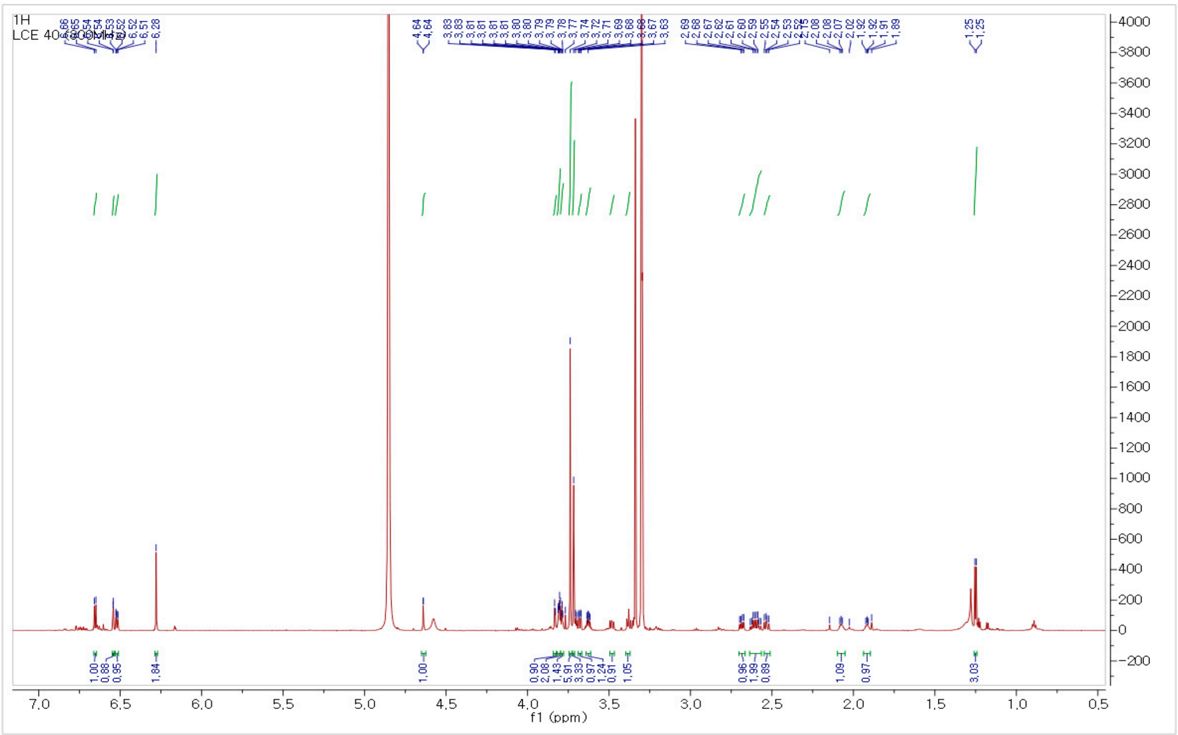

Figure S9. ^{1}H NMR spectrum of compound **2.**

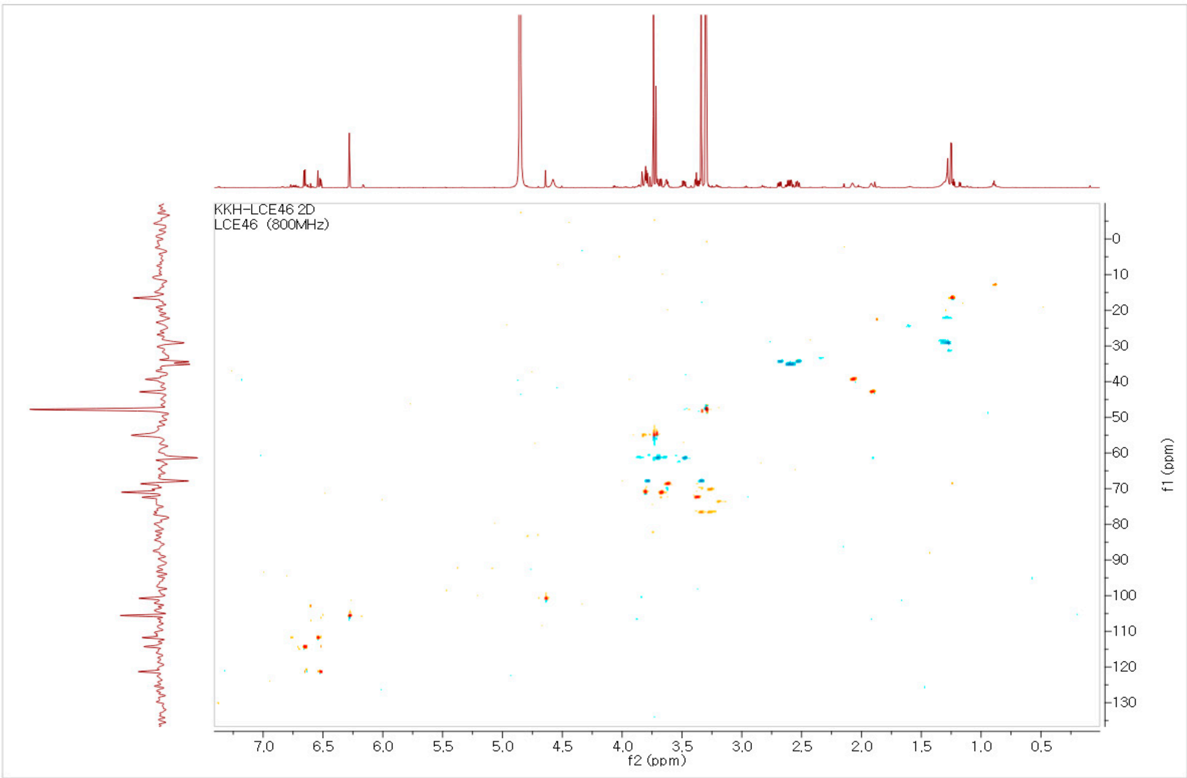

Figure S10. HSQC spectrum of compound **2.**

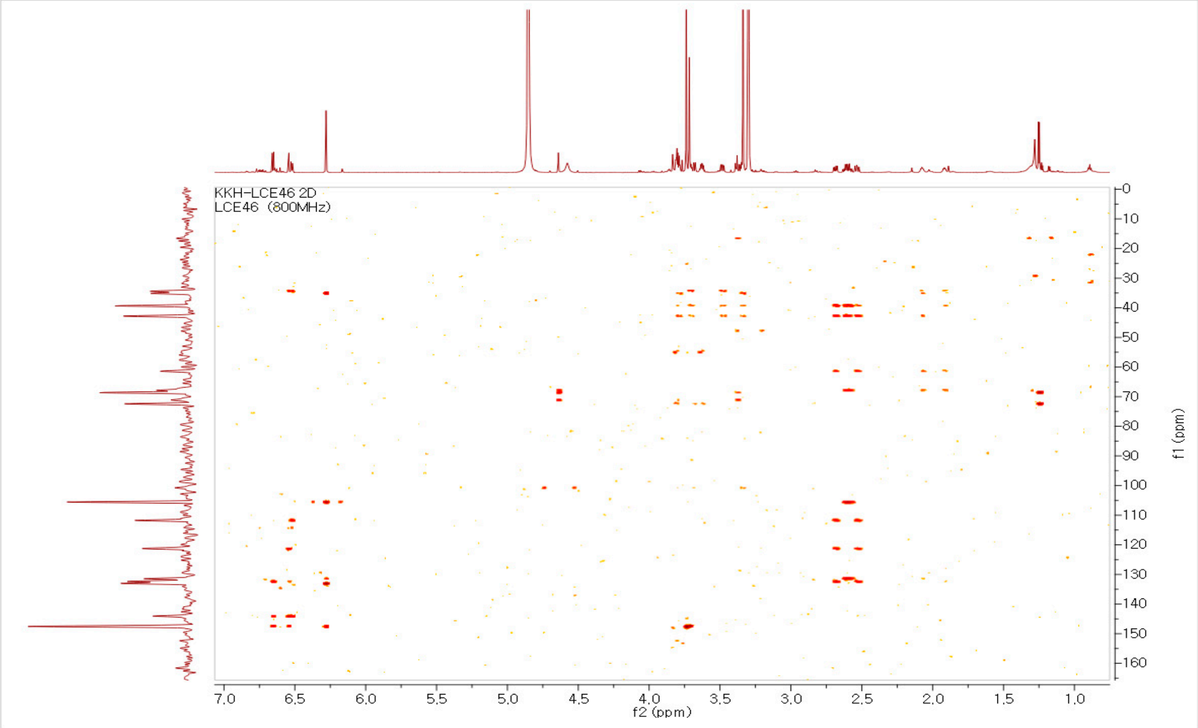

Figure S11. HMBC spectrum of compound **2.**

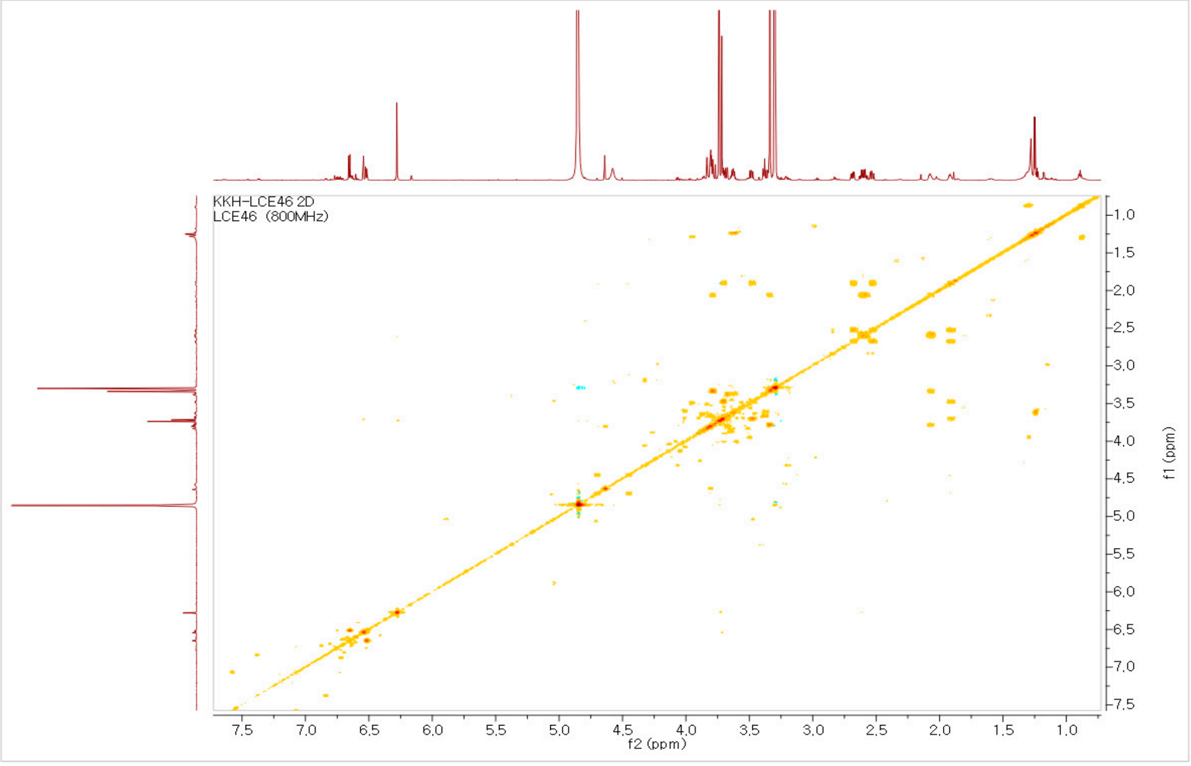

Figure S12. ^{1}H-^{1}H COSY spectrum of compound **2.**

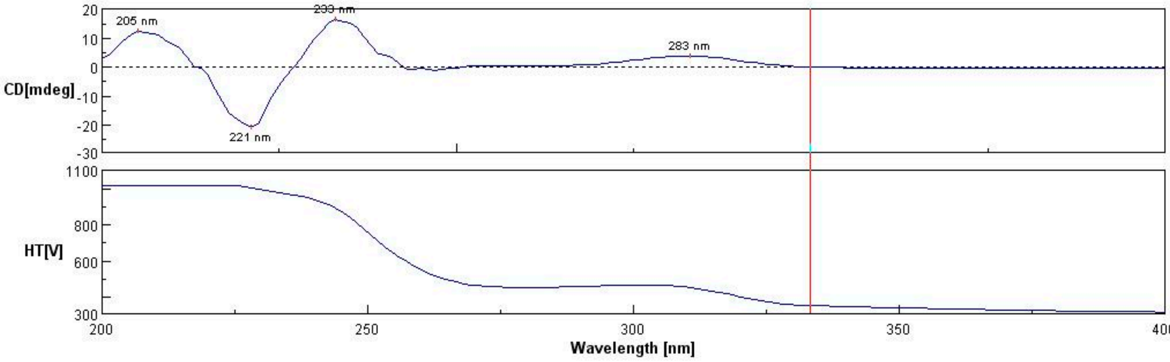

Figure S13. CD spectrum of compound **2.**

LC/MS Analysis of 1 and 2

Stock solutions of the compounds **1** and **2** were prepared by dissolving 0.1 mg of sample in 0.1 mL methanol. Each solution was further diluted with methanol to provide a solution of 100 μg/mL. The solutions were filtered through a 0.45 mm hydrophobic PTFE filter and analyzed by LC/MS (Agilent Technologies, Santa Clara, CA, USA) using a LC-MS Agilent 1200 Series analytical system equipped with a photodiode array (PDA) detector combined with a 6130 Series ESI mass spectrometer. Analysis was performed by injection of 10 μL of each sample using a Kinetex C18 column (2.1 × 100 mm, 5 μm; Phenomenex, Torrance, CA, USA) set at 25°C. The mobile phase consisting of formic acid in H_2O [0.1% (v/v)] (A) and methanol (B) was delivered at a flow rate of 0.3 mL/min by applying the following programmed gradient elution: 10%-100% (B) for 30 min, 100% (B) for 1 min, 100% (B) isocratic for 10 min, and then 10% (B) isocratic for 10 min, to perform post-run reconditioning of the column.

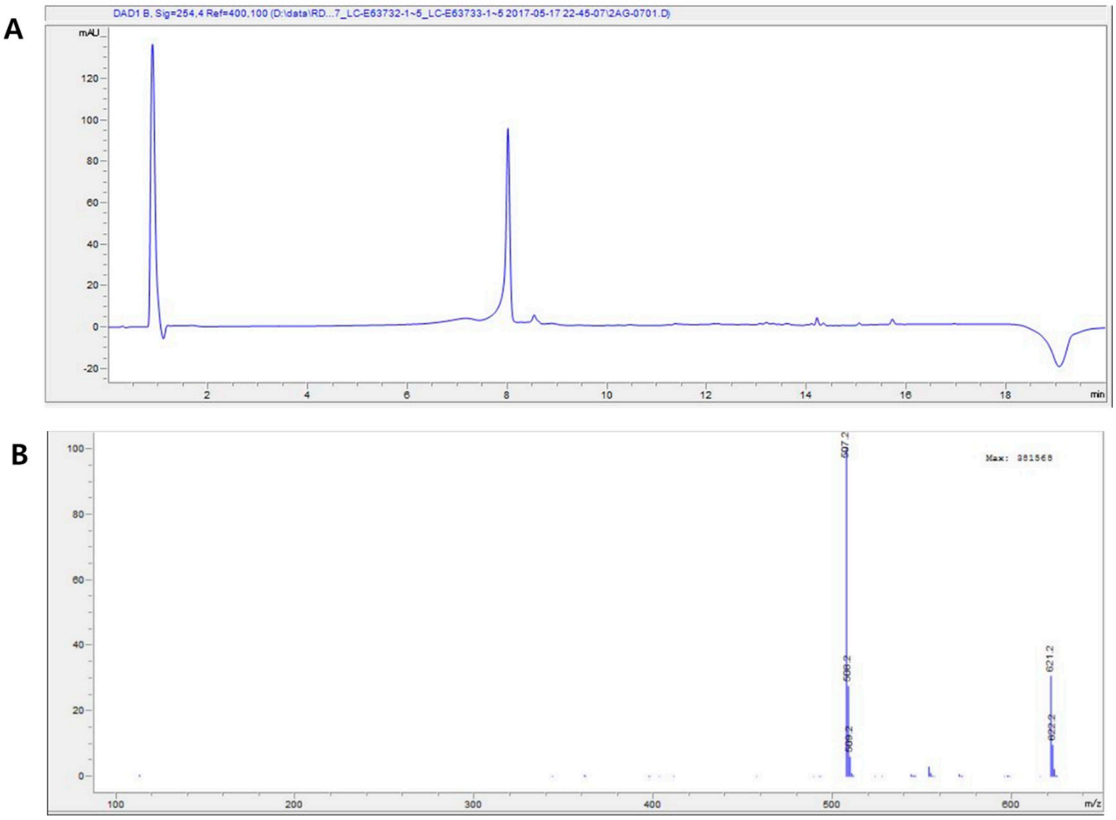

Figure S14. The LC/MS analysis of compound **1**. (**A**) UV chromatogram of LC/MS (detection wavelength was set as 254 nm) of compound **1**. (**B**) Negative ion-mode ESI-MS data of compound **1** in LC/MS analysis.

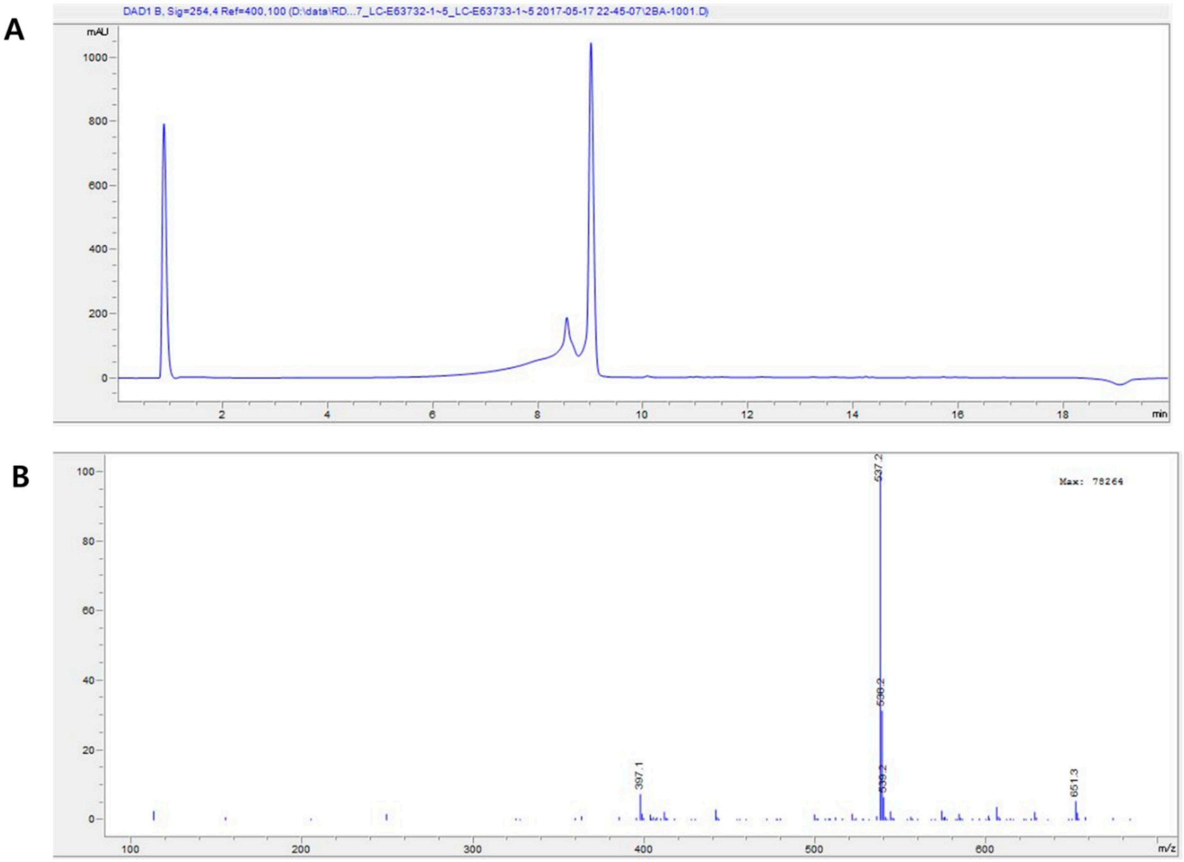

Figure S15. The LC/MS analysis of compound **2**. (**A**) UV chromatogram of LC/MS (detection wavelength was set as 254 nm) of compound **2**. (**B**) Negative ion-mode ESI-MS data of compound **2** in LC/MS analysis.

Table S1 Cytotoxicity of compounds **1**–**14** against four cultured human breast cancer cells using the SRB bioassay in vitro.

Compound	IC_{50} (μM) [a]			
	Bt549	MCF7	MDA-MB-231	HCC70
**1**	26.16	> 30.0	24.38	25.04
**2**	> 30.0	> 30.0	> 30.0	> 30.0
**3**	> 30.0	> 30.0	> 30.0	> 30.0
**4**	> 30.0	28.08	> 30.0	> 30.0
**5**	> 30.0	> 30.0	> 30.0	24.81
**6**	> 30.0	29.18	27.57	28.94
**7**	> 30.0	> 30.0	> 30.0	> 30.0
**8**	> 30.0	> 30.0	> 30.0	> 30.0
**9**	> 30.0	> 30.0	> 30.0	> 30.0
**10**	> 30.0	> 30.0	> 30.0	> 30.0
**11**	> 30.0	> 30.0	> 30.0	> 30.0
**12**	> 30.0	> 30.0	> 30.0	> 30.0
**13**	> 30.0	> 30.0	> 30.0	> 30.0
**14**	> 30.0	> 30.0	> 30.0	> 30.0
Etoposide [b]	1.93	1.12	1.36	1.79

[a] IC_{50} value of compounds against each tumor cell line, which was defined as the concentration (μM) that caused 50% inhibition of cell growth in vitro. [b] Etoposide served as a positive control.
